# Generative AI as a conditional job resource under job demands in academic knowledge work: directed content analysis using the job demands–resources framework

**DOI:** 10.3389/frai.2026.1774525

**Published:** 2026-03-09

**Authors:** Mehmed Zahid Çögenli

**Affiliations:** Department of Occupational Health and Safety, Faculty of Health Sciences, Uşak University, Usak, Türkiye

**Keywords:** academic knowledge work, boundary conditions, directed qualitative content analysis, generative AI, governance, human–AI interaction, job demands–resources model, occupational health psychology

## Abstract

**Background:**

Generative artificial intelligence (GenAI) is rapidly entering knowledge work, yet organizational psychology lacks a clear account of when and how GenAI functions as a job resource in the Job Demands–Resources (JD-R) model, especially in high-demand academic work.

**Methods:**

We conducted a JD-R–guided directed qualitative content analysis of a de-identified human–AI interaction log generated during routine academic work. The log was segmented into interactional episodes and coded using function-first descriptors. Codes were mapped deductively to three JD-R–aligned resource domains (cognitive; structural/strategic; emotional/psychosocial) and a boundary-conditions stream (human oversight; data integrity/traceability).

**Results:**

Across 15 episodes, GenAI enacted cognitive functions that reduced informational complexity, structural/strategic functions that increased planning capacity and task structure, and emotional/psychosocial functions expressed through observable efficacy-reinforcing and action-orienting cues. Boundary-condition coding showed that benefits depended on human oversight and integrity routines; in episodes requiring substantial verification or traceability work, GenAI could shift rather than reduce demands.

**Conclusion:**

GenAI can operate as a conditional job resource in demanding academic knowledge work, but sustainable benefit requires explicit human-in-the-loop oversight and data integrity practices that support reliable and responsible use.

## Introduction

Academic work is increasingly shaped by intensified and competing job demands, including publication pressure, heavy teaching responsibilities, and administrative workload. Evidence on academics’ working conditions shows that such demands accumulate over time and relate to strain and reduced work–life balance, motivating research on how knowledge workers cope with sustained demands (Kinman and Jones, 2008; Naidoo-Chetty and du Plessis, 2021).

This study uses the Job Demands–Resources (JD-R) model to conceptualize coping under demanding work conditions (Bakker and Demerouti, 2007; Demerouti et al., 2001). In JD-R theory, job demands require sustained effort and carry physiological or psychological costs, whereas job resources support goal attainment, reduce demand-related costs, and foster learning and growth (Bakker and Demerouti, 2017). A central proposition is that resources can buffer the negative effects of high demands, making context-relevant resources particularly important in demand-laden work environments (Bakker and Demerouti, 2007). For reader orientation, a redrawn overview of the JD-R model is provided in Supplementary Figure S1.

Against this background, GenAI tools have prompted debate about opportunities, risks, and governance concerns in research and organizational settings (Dwivedi et al., 2023). From an organizational psychology and human–AI interaction perspective, a more specific question remains underdeveloped: can GenAI function as a job resource under job demands, and under what conditions does that support remain practically usable? A JD-R lens shifts attention from GenAI as a technology to GenAI as a set of enacted functions that may operate as resources in practice.

In principle, GenAI could provide cognitive support by reducing informational complexity, structural/strategic support by structuring work and planning, and emotional/psychosocial support via cues that reinforce efficacy or translate uncertainty into actionable steps. However, resource value is plausibly conditional. The same interactions may introduce or shift demands through verification labor, governance constraints, and the need for calibrated trust in automation (Amershi et al., 2019; Lee and See, 2004). These dynamics align with evidence that digital tools can enable performance while generating technostressors when governance and support are insufficient (Ayyagari et al., 2011; Tarafdar et al., 2007).

Recent field and experimental studies suggest that GenAI can raise productivity and sometimes improve quality, with heterogeneous effects across workers and tasks. Gains may be offset by uncertainty about accuracy and the oversight required to manage failures (Brynjolfsson et al., 2025; Noy and Zhang, 2023). These patterns motivate episode-level analysis of how GenAI support is enacted in real interaction sequences and under which boundary conditions it stabilizes as a net resource rather than a demand-shifting mechanism.

To address this gap, we conceptualize GenAI as a potential conditional job resource and examine how resource-like functions are enacted under job demands using a JD-R–guided directed qualitative content analysis (Hsieh and Shannon, 2005) of a de-identified human–AI interaction log generated by an academic knowledge worker. We ask: In human–AI interactions occurring under job demands, what functional roles does generative AI enact that can be categorized as job resources within the JD-R framework, and what boundary conditions shape the practical usability of these resources?

We contribute to the literature in three ways:

- We specify a JD-R–aligned, function-first typology of AI-mediated job resources (cognitive; structural/strategic; emotional/psychosocial).
- We formalize socio-technical boundary conditions—human-in-the-loop (HITL) oversight and data integrity/traceability routines—as determinants of whether GenAI reduces demands or shifts demands to verification work.
- We clarify the scope of inference by framing the design as a single-case analysis aimed at analytic generalization (mechanism specification), not empirical or statistical generalization.

## Materials and methods

### Study design

This study employed a directed qualitative content analysis approach (Hsieh and Shannon, 2005) to classify the functional roles enacted by a generative AI system within a human–AI interaction log. A directed approach is appropriate when an existing theoretical framework guides category development and deductive mapping. Accordingly, analysis was guided by the JD-R model (Bakker and Demerouti, 2007; Bakker and Demerouti, 2017; Demerouti et al., 2001).

The design is a single-case, single-user qualitative study. Its purpose is to specify candidate mechanisms and boundary conditions through analytic generalization rather than estimate prevalence or causal effects (Yin, 2018).

### Data source and context

The dataset consisted of an exported transcript of human–AI interactions produced during routine academic work by a university faculty member. The content spans work-related prompts (e.g., academic writing, task structuring, decision support) and contextual constraints that shaped the work period, including health-related constraints referenced in the log. Health-related references are treated as contextual constraints rather than analytic targets.

The transcript captures multiple prompt–response sequences between a single user (the author) and a generative AI chatbot (Google Gemini; accessed 8–12 November 2025). After removing platform boilerplate, the cleaned transcript contained 464 non-empty paragraphs and approximately 7,846 words.

### Unit of analysis and episode inclusion

The unit of analysis was the interactional episode, defined as a prompt–response sequence (one user prompt and the immediately corresponding AI output). Where a single user prompt produced a multi-part AI output (e.g., numbered steps or structured recommendations), the full output was treated as part of the same episode to preserve functional coherence.

Episodes were included if they directly contributed to (a) sensemaking, (b) planning or task structuring, (c) psychosocially relevant support expressed through observable output cues, or (d) explicit boundary-condition management in the focal workflow. Non-substantive exchanges (e.g., greetings or acknowledgments) were removed during cleaning. Episodes were segmented to preserve functional coherence when consecutive turns addressed the same goal and constraint set.

The final set of 15 episodes reflects all functionally relevant episodes in the focal workflow after removing non-substantive turns; as a single-case design, adequacy is judged by conceptual coverage of enacted functions and boundary conditions rather than statistical representativeness.

### Data preparation and de-identification

Because the transcript contained potentially sensitive personal and health-related information, the dataset was de-identified prior to analysis. Direct identifiers (names, locations, institutional identifiers, and other uniquely identifying references) were removed or generalized. Each interactional episode was assigned an episode ID (e.g., E01, E02) to support transparent tracking from coded excerpts to analytic claims without exposing identifying content.

### Analytic framework and coding categories (JD-R–guided)

Coding was guided by the JD-R definition of job resources as aspects that (a) support goal achievement, (b) reduce the costs of job demands, and/or (c) foster learning and development (Bakker and Demerouti, 2017; Demerouti et al., 2001). The analytic objective was to classify AI-enacted functions as resource-like roles.

We operationalized three categories of AI-mediated job resources: (1) cognitive job resources (functions that reduce informational complexity and support comprehension and decision-oriented sensemaking, such as explanation, clarification, summarization, and reframing), (2) structural/strategic job resources (functions that enable planning, sequencing, and task structuring, such as roadmaps, checklists, procedural structuring, formatting guidance, and work organization), and (3) emotional/psychosocial job resources (functions that support perceived self-efficacy and action orientation through observable cues). In addition, we coded boundary conditions shaping resource effectiveness, especially where interaction invoked verification practices, human oversight, or data integrity requirements.

To preserve the function-first stance, emotional/psychosocial resources were identified strictly through functionally observable cues in the AI output (e.g., explicit efficacy reinforcement paired with concrete next steps), not by inferring user affect or outcomes.

### Coding procedure

The directed content analysis proceeded in four steps (Hsieh and Shannon, 2005):

1. Familiarization and episode segmentation: The transcript was reviewed end-to-end and segmented into interactional episodes; non-conversational boilerplate was removed.
2. Function-first descriptive coding: Each AI output was coded using verb-oriented functional descriptors (e.g., explains, structures, summarizes, reframes, requests verification), avoiding experiential interpretation.
3. Deductive mapping to JD-R resource categories: Descriptive codes were mapped to the three resource domains using JD-R criteria (goal support, demand-cost reduction, and support for effective functioning under demands).
4. Boundary-condition coding and consolidation: Episodes indicating constraints (e.g., verification needs, reliability limitations, traceability requirements) were coded as boundary conditions. Category definitions were refined to improve consistency while allowing multi-label coding when distinct functions co-occurred in the same episode.

A coding matrix documented episode IDs, functional descriptors, JD-R category assignments, and boundary-condition coding. Exemplar episodes are summarized in Table 1; the full matrix is provided in Supplementary Table S1.

**Table 1:** Episode-level mapping of AI-enacted functions to JD-R resource categories and boundary conditions.

Category	Operational definition	Core AI-enacted functions (examples)	Exemplar episodes
Cognitive job resources	Functions that reduce informational complexity and support comprehension/sensemaking	Concept clarification; mechanism-to-outcome explanation; report-style interpretation; decision-relevant framing	E01–E04, E10
Structural/strategic job resources	Functions that increase control, predictability, and planning capacity under demands	Roadmaps and sequencing; decision-path structuring; prioritization; work organization and deliverable planning	E05–E06, E08–E11
Emotional/psychosocial job resources	Functions that support perceived self-efficacy and action orientation through observable cues	Efficacy reinforcement; controllability-based reframing paired with actionable steps	E07 (cues in E08–E11)
Boundary conditions	Safeguards/constraints shaping reliability and practical usability	Data integrity/traceability workflows; explicit correction and revision (human oversight)	E13–E15

HITL, human-in-the-loop (explicit user oversight, correction, or verification of AI outputs).

Data integrity/traceability, procedures for reliable capture, storage, and reproducibility of interaction records. Mappings reflect episode-level functional interpretation and are intended for analytic generalization, not statistical generalization.

### Analytic transparency and trustworthiness

To enhance transparency, an audit trail was maintained that recorded segmentation rules, category definitions, decision rules, and category refinements (Hsieh and Shannon, 2005). A second-pass review was conducted after a two-week time lag to check stability of coding and to reduce overgeneralization. In particular, emotional/psychosocial resources were coded only when functionally present in the AI output rather than inferred.

A code–recode procedure was conducted after a two-week time lag to assess stability of functional codes and JD-R mappings. Discrepancies were resolved through rule refinement and memoing, and the final decision rules are reflected in the audit trail (Supplementary Table S1). Negative or countervailing evidence (e.g., verification burden, reliability constraints) was retained via boundary-condition coding rather than forced into a resource interpretation.

Positionality and dual-role management: Because the author served as both researcher and data source, analytic choices prioritized interpretive discipline and privacy protection. The analysis focused on observable AI-enacted functions (function-first coding) rather than claims about the author’s experiences or outcomes. De-identification and episode-level tracking were used to reduce re-identification risk while preserving an inspectable audit trail.

### Use of AI tools during analysis (disclosure)

No generative AI tools were used to generate codes or make category assignments. Coding and categorization were performed by the author using the de-identified transcript and a manually maintained coding matrix.

## Results

### Overview of the coded dataset

**Figure 1:**
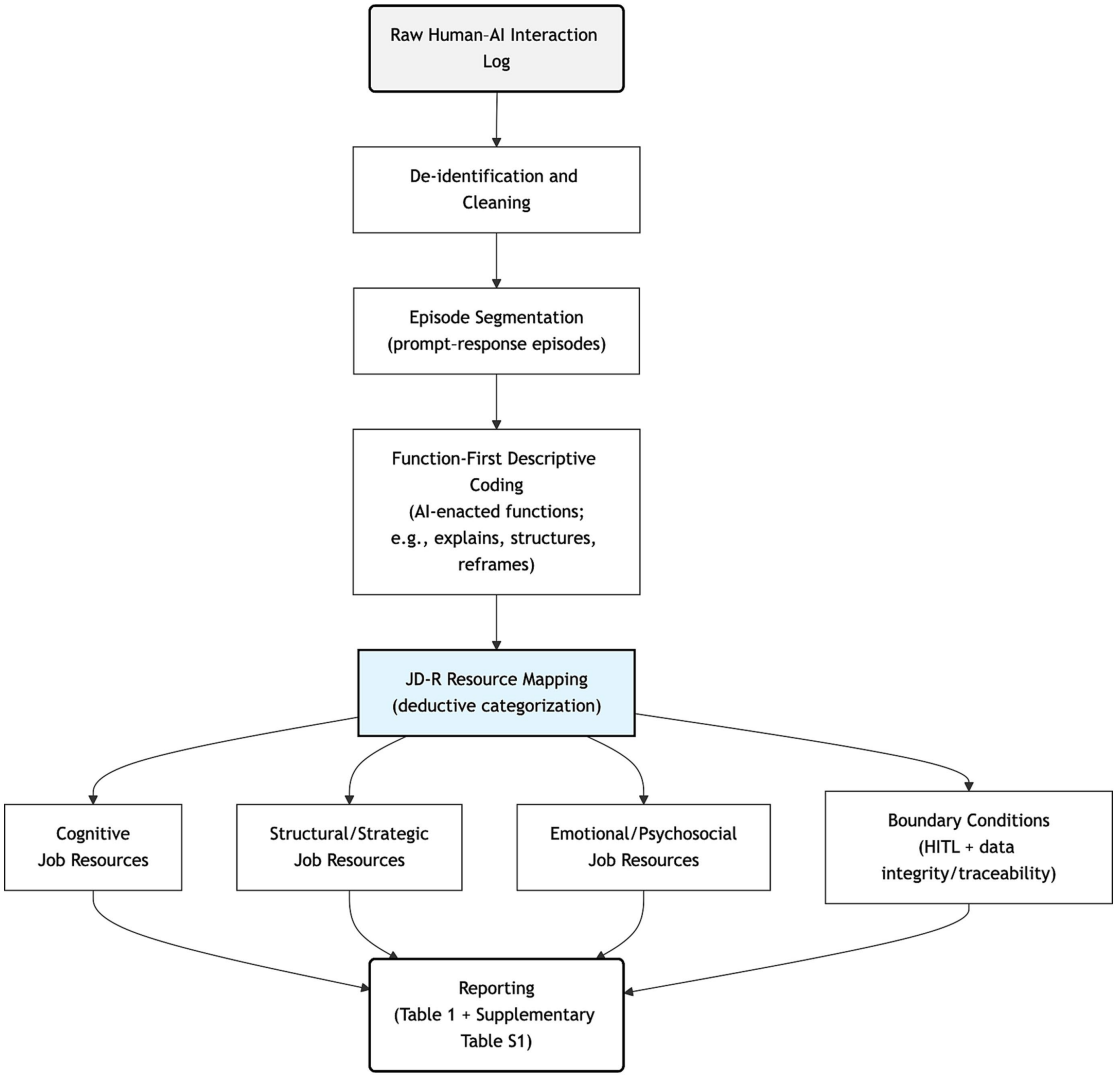
Directed content analysis workflow and JD-R mapping. The raw human–AI interaction log was de-identified and cleaned, segmented into prompt–response episodes, and coded using function-first descriptive codes (e.g., explaining, structuring, reframing). Descriptive codes were then deductively mapped to JD-R resource domains (cognitive; structural/strategic; emotional/psychosocial) and boundary conditions (human-in-the-loop, HITL; and data integrity/traceability). Reporting draws on Table 1 and Supplementary Table S1.

The dataset comprised 15 interactional episodes (E01–E15), each defined as a user prompt and the immediately corresponding AI output(s). Using a JD-R–guided directed content analysis, AI-enacted functions were mapped deductively to job resource domains. The analytic focus was functional: what the AI did across episodes, rather than experiential interpretation. Functions clustered into cognitive, structural/strategic, and emotional/psychosocial job resources, alongside boundary conditions (Table 1; Figure 1; Supplementary Table S1).

### Cognitive job resources

Cognitive job resources were operationalized as AI functions that reduced informational complexity and supported comprehension and decision-oriented sensemaking under job demands. These functions appeared in episodes where the user sought clarification of concepts, translation of specialized terms into accessible reasoning, and structured interpretation of report-like information. The AI often transformed complex information into decision-relevant framing and explicitly structured uncertainty into actionable logic (Supplementary Table S1).

Exemplar episodes for cognitive resources: E01–E04 and E10.

### Structural/strategic job resources

Structural/strategic job resources were operationalized as functions that increased control, predictability, and planning capacity under demands. These functions were evident when interaction shifted from information-seeking to action structuring, including sequencing tasks, organizing options into coherent pathways, and generating roadmaps for next steps. In several episodes, the AI supported prioritization by proposing non-overlapping deliverables and timeline-oriented work organization (Supplementary Table S1).

Exemplar episodes for structural/strategic resources: E05–E06 and E08–E11.

### Emotional/psychosocial job resources

Emotional/psychosocial job resources were operationalized as AI functions that supported perceived self-efficacy and action orientation through observable output cues. In the dataset, these functions most commonly appeared as efficacy reinforcement and controllability-based reframing paired with concrete next actions. Consistent with the function-first stance, coding was limited to outputs where such cues were explicit rather than inferred (Supplementary Table S1).

Exemplar episodes for emotional/psychosocial resources: E07 (with supportive cues in E08–E11).

### Boundary conditions and human oversight requirements

Boundary conditions were operationalized as safeguards and constraints shaping when AI outputs could be treated as reliable and practically usable. These conditions were most visible in episodes focused on data integrity and traceability (complete export, archiving, and reproducibility of interaction records) and in episodes requiring explicit correction and revision following contextual mismatch. These patterns indicate that GenAI operates as a conditional job resource: usefulness depends on human oversight practices such as verification, correction, and reliable record handling (Supplementary Table S1).

Exemplar episodes for boundary conditions: E13–E15.

Table 1 summarizes the JD-R–aligned typology and episode distribution, while Supplementary Table S1 provides the full episode-level audit trail.

## Discussion

### Principal findings

This study examined whether and how GenAI can operate as a job resource in demanding academic knowledge work. Directed content analysis of a de-identified interaction log indicates that GenAI enacted resource-like functions across cognitive, structural/strategic, and emotional/psychosocial domains, while also revealing boundary conditions that qualify when these functions translate into net benefit (Table 1; Figure 1; Supplementary Table S1).

Findings should be interpreted as an episode-level functional typology. The study does not test JD-R health-impairment or motivational pathways; instead, it specifies candidate mechanisms and boundary conditions through which GenAI may be enacted as a conditional job resource under high demands. Any links to engagement, strain, or performance remain theoretical propositions rather than empirically tested outcomes in this dataset.

### Theoretical implications: AI-mediated support as JD-R resources

A central implication is that GenAI can instantiate a non-human analogue of support typically studied as social or informational resources in JD-R research. The data show AI-mediated cognitive support (clarification, explanation, complexity reduction) and structural/strategic support (sequencing, roadmaps, task structure) that plausibly reduce friction and uncertainty in knowledge-intensive work. A further implication concerns emotional/psychosocial support: when delivered as explicit efficacy-reinforcing cues paired with concrete next steps, GenAI outputs may support perceived control during demanding periods.

Conceptually, the resource value of GenAI is not a fixed property of the tool. It is an enacted set of functions shaped by the interaction sequence, the user’s work context, and the quality-control environment. This framing clarifies why GenAI is better treated as a conditional job resource in JD-R terms.

### Practical implications: governance and inclusive work design

The findings suggest that GenAI may function as assistive support by reducing operational friction in complex tasks through cognitive offloading and work structuring (Risko and Gilbert, 2016). In high-demand periods, such support may help users translate ambiguity into actionable plans. However, the same interactions can shift demands onto users through verification labor and the need to calibrate reliance on uncertain outputs, especially in high-stakes knowledge work (Amershi et al., 2019; Lee and See, 2004).

Accordingly, organizations considering GenAI in knowledge work should treat verification norms, accountability allocation, and integrity/traceability routines as core elements of responsible implementation. Without these scaffolds, the effort required for correction and documentation can become an additional demand that partially negates resource gains.

### Boundary conditions: why GenAI is a conditional job resource

Boundary-condition coding indicates that resource benefits depended on human oversight and data integrity/traceability practices. When oversight routines were strong, AI outputs were more readily usable for sensemaking and planning. When oversight and traceability work increased, GenAI could shift rather than reduce demands. This pattern suggests a potential resource paradox: weak governance can convert apparent resource gains into additional demands via verification, correction, and accountability work.

Practically, this implies that AI literacy and governance capacity should be treated as part of work design rather than assumed automation savings.

### Limitations and future research

The study is limited by its single-user, single-context design, which constrains analytic breadth and raises the possibility of idiosyncratic interaction patterns. The author’s role, expertise, and interaction style may have shaped which functions were elicited and how prompts were framed. Therefore, the typology is intended for analytic generalization (mechanism specification), not empirical generalization to populations or occupations.

Future research should examine multi-user corpora across roles and occupations, test links between AI-enacted resources and JD-R outcomes using mixed methods and longitudinal designs, and explicitly model verification overhead and integrity work as moderators that determine when GenAI becomes a net resource versus a demand shift.

## Conclusion

By mapping AI-enacted functions to JD-R–aligned resource domains and specifying oversight- and integrity-related boundary conditions, this study advances a grounded account of GenAI as a conditional job resource. GenAI can provide cognitive, structural/strategic, and emotional/psychosocial resources (Table 1; Figure 1; Supplementary Table S1), but the extent to which these translate into sustainable benefit depends on human-in-the-loop oversight and data integrity/traceability practices that secure reliability and responsible use.

## Data Availability

The original interaction transcript contains sensitive personal and health-related information and is therefore not publicly available. De-identified, episode-level materials supporting the findings are provided in Supplementary Table S1. A redrawn overview of the JD-R model is provided as Supplementary Figure S1. Additional access requests will be considered by the corresponding author on a case-by-case basis, subject to privacy and data-protection constraints.
