# Supplementary figures and images for: Generative AI as a conditional job resource under job demands in academic knowledge work: directed content analysis using the job demands–resources framework
Source: Front Artif Intell. 2026 Mar 9;9:1774525. doi: 10.3389/frai.2026.1774525 (PMC13006688; doi:10.3389/frai.2026.1774525)

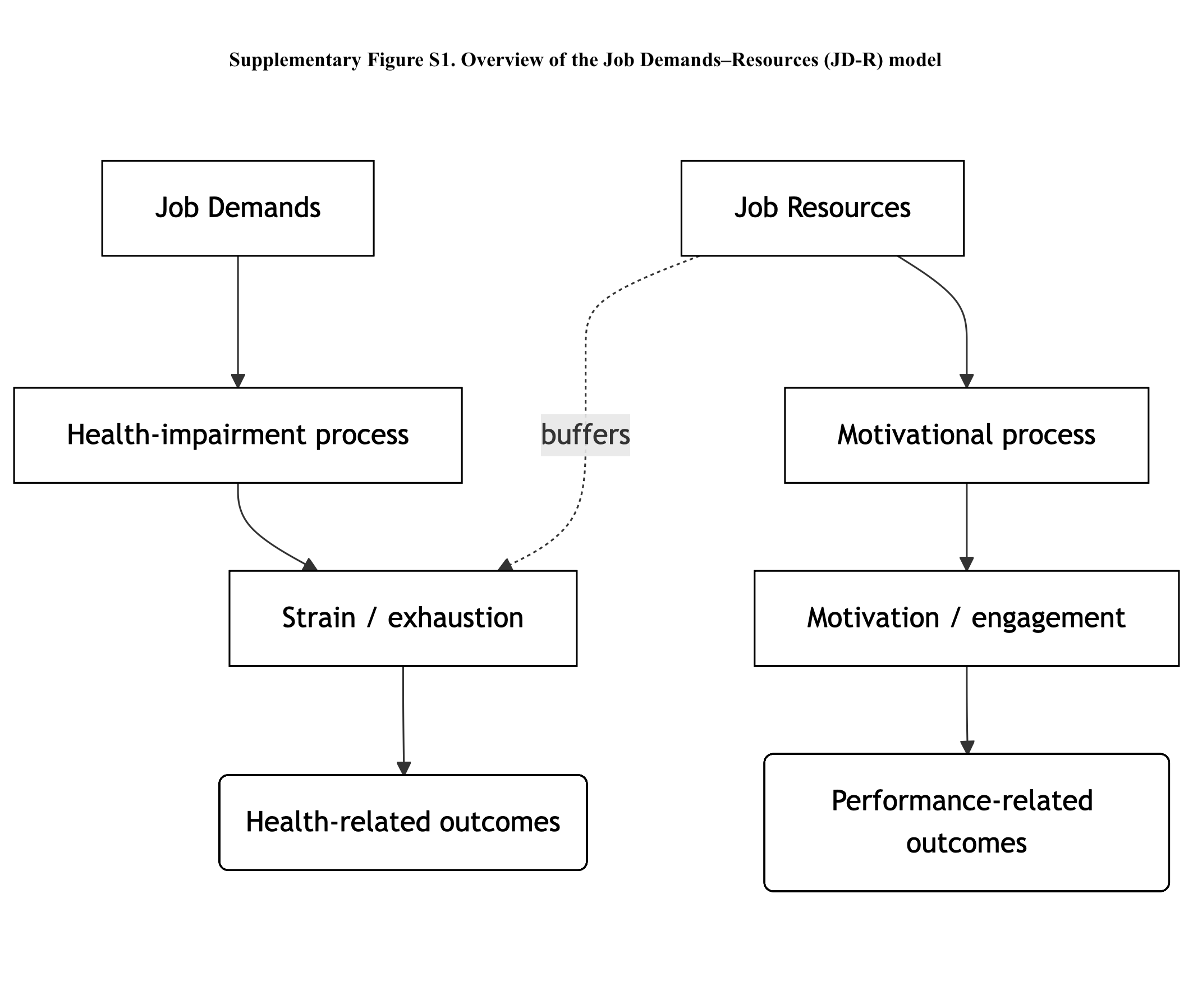

Supplement: Supplementary file 1 [file Image_1.PNG]
