# Supplementary material for: Generative AI as a conditional job resource under job demands in academic knowledge work: directed content analysis using the job demands–resources framework
Source: Front Artif Intell. 2026 Mar 9;9:1774525. doi: 10.3389/frai.2026.1774525 (PMC13006688; doi:10.3389/frai.2026.1774525)
Supplement: Supplementary file 2 [file Table_1.DOCX]

**Supplementary Table S1 (Data Sheet 1). Episode-level audit trail for JD-R–guided directed content analysis.** For each interactional episode (E01–E15), the table provides a de-identified English paraphrase, functional labels (AI-enacted functions), and the corresponding JD-R mapping. Health-related references were generalized to reduce identifiability; the table uses de-identified English paraphrases, not verbatim excerpts.

| Episode | User prompt (EN paraphrase) | AI output summary (EN paraphrase) | Function label  (what AI does) | Mapping |
| --- | --- | --- | --- | --- |
| E01 | User describes living with a chronic neuromuscular condition and asks for understanding | Explains the condition conceptually (neuropathy framing), links mechanisms to functional implications, and provides practical management considerations | Concept clarification  + mechanism explanation  + practical guidance | Cognitive (primary); Structural (secondary) |
| E02 | User asks whether it is genetic | Explains hereditary transmission patterns and distinguishes major inheritance modes | Inheritance-structure explanation | Cognitive |
| E03 | User asks about probability of transmission to children | Translates inheritance logic into decision-relevant probabilities and clarifies implications | Probability translation  + implication framing | Cognitive |
| E04 | User asks whether symptoms can be absent and whether there are rates | Explains variability mechanisms and provides a structured framing of severity variability | Variability framing  + uncertainty management | Cognitive |
| E05 | User describes relatives and asks implications | Organizes family information into a structured risk map and clarifies how risk differs across relatives | Risk-map structuring | Structural/strategic |
| E06 | User asks “what next?” | Provides a staged roadmap and outlines option pathways in a sequential manner | Multi-step roadmap  + decision-path structuring | Structural/strategic |
| E07 | User expresses desire to be a good example | Reinforces self-efficacy and reframes uncertainty toward controllable actions | Efficacy reinforcement  + controllability reframing | Emotional/psychosocial |
| E08 | User wants to continue with questions | Provides a structured “question framework” and organizes next inquiry routes | Interaction structuring  + guided questioning | Structural/strategic (primary) |
| E09 | User asks if multiple outputs/publications are possible | Structures a non-overlapping deliverable plan and outlines how to package outputs | Deliverable planning  + sequencing | Structural/strategic |
| E10 | User asks whether other family branches can have the same report result | Clarifies how the same genetic result could appear across branches and how to reason about it | Cross-branch interpretation  + logic clarification | Cognitive (primary); Structural (secondary) |
| E11 | User asks for a bottom-line synthesis (“so what?”) | Provides a two-track synthesis: (a) practical next steps and (b) structured work/goal roadmap | Synthesis + prioritization roadmap | Structural/strategic |
| E12 | User says they do not want to ignore the AI’s help | Provides guidance on responsible acknowledgment and boundaries without centering authorship claims | Responsible-use framing  + boundary setting | Boundary condition (light); Structural (secondary) |
| E13 | User asks how to save the whole conversation | Provides an end-to-end workflow to preserve the full log reliably | Data integrity workflow | Boundary condition |
| E14 | User reports export captures only the last response | Diagnoses platform limitation and gives a reproducible workaround for complete capture | Data integrity troubleshooting | Boundary condition |
| E15 | User corrects an identity/context detail | Acknowledges the mismatch and revises the approach accordingly | Explicit correction  + revision (HITL) | Boundary condition |
